# Emerging issues in probiotic safety: 2023 perspectives

**DOI:** 10.1080/19490976.2023.2185034

**Published:** 2023-03-15

**Authors:** Daniel Merenstein, Bruno Pot, Gregory Leyer, Arthur C. Ouwehand, Geoffrey A. Preidis, Christopher A. Elkins, Colin Hill, Zachery T. Lewis, Andi L. Shane, Niv Zmora, Mariya I. Petrova, Maria Carmen Collado, Lorenzo Morelli, Gina A. Montoya, Hania Szajewska, Daniel J. Tancredi, Mary Ellen Sanders

**Affiliations:** a Department of Family Medicine, Georgetown University Medical Center, Washington, DC USA; b Yakult Europe BV, Almere, Netherlands; c Scientific Affairs, Chr. Hansen, Milwaukee, WI, USA; d Global Health & Nutrition Sciences, International Flavors & Fragrances, Kantvik, Finland; e Division of Gastroenterology, Hepatology & Nutrition, Department of Pediatrics, Baylor College of Medicine and Texas Children’s Hospital, Houston, TX, USA; f Clinical and Environmental Microbiology Branch, Division of Healthcare Quality Promotion, National Center for Emerging and Zoonotic Infectious Diseases, Centers for Disease Control and Prevention, Atlanta, GA, USA; g APC Microbiome Ireland, University College Cork, Cork, Ireland; h Synbiotic Health Inc, Lincoln, NE, USA; i Division of Infectious Diseases, Department of Pediatrics, Emory University School of Medicine and Children’s Healthcare of Atlanta, Emory Children’s Center, Atlanta, Georgia; j Scientific consultant, Elinav Lab, Immunology Department, Weizmann Institute of Science, Department of Gastroenterology and Liver Diseases, Tel Aviv, Israel; k Winclove Probiotics B.V., Amsterdam, The Netherlands; l Institute of Agrochemistry and Food Technology-National Research Council (IATA-CSIC), Valencia, Spain; m Department of Food Science and Technology, Università Cattolica del Sacro Cuore, Piacenza, Italy; n Department of Chemical Risk Assessment, Nestlé S.A., Lausanne, Switzerland; o Department of Paediatrics, The Medical University of Warsaw, Warsaw, Poland; p Department of Pediatrics, UC Davis School of Medicine, Sacramento, CA, USA; q International Scientific Association for Probiotics and Prebiotics, Centennial, CO, USA

**Keywords:** Probiotic, safety, next-generation probiotics, antibiotic resistance, microbiome, microbiota, genome sequencing, live biotherapeutic products, International Scientific Association for Probiotics and Prebiotics, ISAPP

## Abstract

Probiotics are used for both generally healthy consumers and in clinical settings. However, theoretical and proven adverse events from probiotic consumption exist. New probiotic strains and products, as well as expanding use of probiotics into vulnerable populations, warrants concise, and actionable recommendations on how to work toward their safe and effective use. The International Scientific Association for Probiotics and Prebiotics convened a meeting to discuss and produce evidence-based recommendations on potential acute and long-term risks, risks to vulnerable populations, the importance for probiotic product quality to match the needs of vulnerable populations, and the need for adverse event reporting related to probiotic use. The importance of whole genome sequencing, which enables determination of virulence, toxin, and antibiotic resistance genes, as well as clear assignment of species and strain identity, is emphasized. We present recommendations to guide the scientific and medical community on judging probiotic safety.

## Plain Language Summary

**What is the context?** Probiotics, available to healthy consumers as both dietary supplements and foods, are also used by some patient populations. The goal of this paper is to determine if any new factors have emerged that would impact current views about probiotic safety for both these populations.

**What is new?** The authors conclude that established practices are sensibly addressing factors important to the safety of traditional probiotics used by the general population. They also make recommendations regarding emerging safety considerations. Probiotics targeted for patient populations should undergo stringent testing to meet quality standards appropriate for that population, preferably verified by an independent third party. The safety of probiotics derived from species without a history of safe use must be considered on a case-by-case basis. Research is needed to address some gaps, for example which best animal models to use for safety assessment of live microbes, the possibility of antibiotic resistance gene transfer via transformation, and potential impact of probiotic-induced changes in microbiomes, interactions with drugs, and probiotic colonization.

**What is the impact?** Probiotics of sufficient quality for patient populations are being developed and should be used accordingly. Long-term safety assessments for probiotics should be consistent with, and not more stringent than, current regulatory requirements for biologic drugs, including fecal microbial transplants. Rigor in collecting and reporting data on adverse events is needed. The authors confirm the need for understanding the entire genetic makeup of a probiotic as a cornerstone for assessing its safety.

## Background

Probiotics have been defined as ‘live microorganisms that, when administered in adequate amounts, confer a health benefit on the host’, a definition that was a grammatical edit of a previous FAO expert consultation.^1^ In the last 20 years, the number and quality of clinical trials assessing the health benefits of probiotics has grown substantially. Advances in efficacy measurement have been integral to the growth of the category. As is the case for any intervention, it is important not just to measure benefits but also to characterize risks. Early probiotics were associated with traditional uses in naturally fermented food products and thus not viewed as drugs, perhaps leading to less attention in earlier probiotic research to adverse event (AE) monitoring and reporting. Among the general population and in patients who are not immunocompromised or severely debilitated, acute safety issues appear to be minor, especially when considering the large global use of probiotics in foods and nutritional supplements.^2–5^ Further, more recent clinical trials reflect much improved reporting of AEs. As is the case with most interventions, though, longer-term safety endpoints are seldom tracked by trialists. As live products, there are theoretical risks of long-term impact on microbiota, immunity, cardiometabolic, and other physiological parameters that deserve further discussion.

The topic of probiotic safety has been addressed by several groups.^6–10^ A foundational initiative by the European Food Safety Authority established the Qualified Presumption of Safety approach for live microorganisms used in foods.^11^ This guidance is useful for recognizing microbial species with a history of safe use in foods. Pariza and colleagues^12^ proposed a 15-step decision tree to guide safety evaluations for products that lack an established history of safe use. An important paper modeled how to evaluate safety at the strain level, applied to the widely used *Enterococcus faecium* SF68.^13^ Roe and colleagues^14^ summarized best practices for assessing the quality and safety of probiotics, noting diverse regulatory frameworks utilized in different global regions.

A highly cited and thorough review of probiotic safety was conducted by the Southern California Evidence-based Practice Center, sponsored by the Agency for Healthcare Research and Quality.^5^ The review conducted in 2009 identified 622 studies, of which only 235 (37%) provided nonspecific statements about safety. Hempel et al. concluded that, from the available evidence, although interventions and AEs were poorly documented, there was no statistically significant increase of the relative risk (RR) of the overall number of experienced AEs (RR 1.00; 95% confidence interval: 0.93, 1.07, *p* = 0.999) associated with probiotic use.

**Figure 1.**
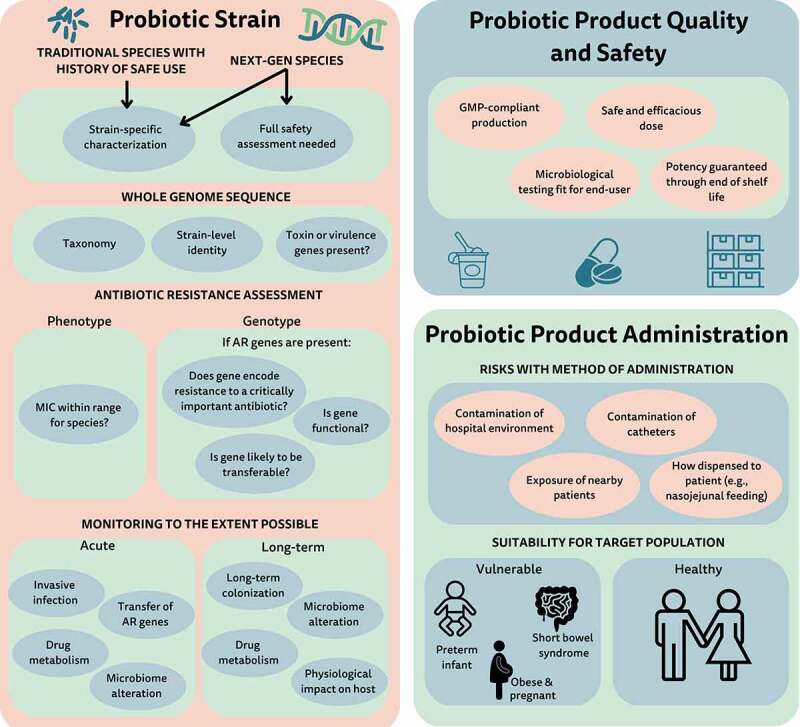
Factors for safety assessment of probiotics. AR, antibiotic resistance; GMP, good manufacturing requirements; MIC, minimum inhibitory concentration; Next-gen, next-generation.

Over the 12 years since the Hempel and colleagues review was published, improved knowledge of the microbiota and probiotics warrants a new look at the question of probiotic safety. This paper is the result of a discussion of an expert panel convened by the International Scientific Association for Probiotics and Prebiotics (ISAPP) at their 2022 annual meeting. ISAPP is a non-profit organization dedicated to advancing the science of probiotics and prebiotics. The assembled panel considered emerging issues pertaining to the safety of probiotics that warrant reconsideration as new data arise (Figure 1). Such issues include potential acute and long-term risks, the need for long-term studies, considerations for vulnerable target populations, probiotic product quality, and the importance of robust reporting of adverse events. The authors met for a half-day to discuss information presented by some panelists leading to a general agreement on key conclusions and recommendations. Individuals authored sections related to their expertise and that text was compiled, reviewed, and edited by all authors.

## Fundamentals of probiotic safety

Concerns related to the safety of probiotic organisms have been raised by clinicians, researchers, and policymakers.^15–17^ These may be broadly characterized into concerns pertaining to the probiotic strain, product quality, or probiotic administration.

A foundation to assessing the safety of any given probiotic strain is a complete genome sequence. This allows assignment of the strain to a given taxonomic group, enabling review of published risks associated with the species. A full genome sequence also allows strain-level identification, which can be important for tracking the strain during production and to investigate etiology of suspected infections. Further, the genome can be interrogated for any genes of concern, including toxigenicity, pathogenicity, or antibiotic resistance (AR). As discussed in more detail below, one concern is the theoretical situation in which probiotic-borne AR genes could be transferred to resident potential pathogens, other microbes harbored by the host, and/or environmental microbes, thereby increasing the ecological pool of antimicrobial resistance genes.^15–17^ Scientists are still evaluating the real transfer risk and the clinical and public health implications of such transfer. Some phenotypic testing is also a component of assessing safety of the probiotic strain.

Issues related to safety pertaining to formulation of probiotic products include the need to establish purity, potency (the quantity of live microbes delivered), and composition of the final product. Further, probiotic products must undergo adequate testing – adapted to the intended use – for potential contaminants.^15–17^ Of particular concern is the presence of unwanted live microbial contaminants. Since probiotics are designed to be administered as live microbes, contamination with pathogenic or potentially pathogenic microbes is a greater risk than for products that undergo an intentional sterilization process. Testing specifications can be tailored for products, with products destined for more vulnerable populations undergoing testing that is more stringent than those used for the general population (Table 1). Microbial contamination of the final product and the presence of allergens or other contaminants are also concerns, but no more so for probiotics than for any other intervention.

**Table 1.** Example testing requirements for a given probiotic developed for different product types and target populations. Note that stringent testing requirements can be imposed for products targeted for the most vulnerable populations.

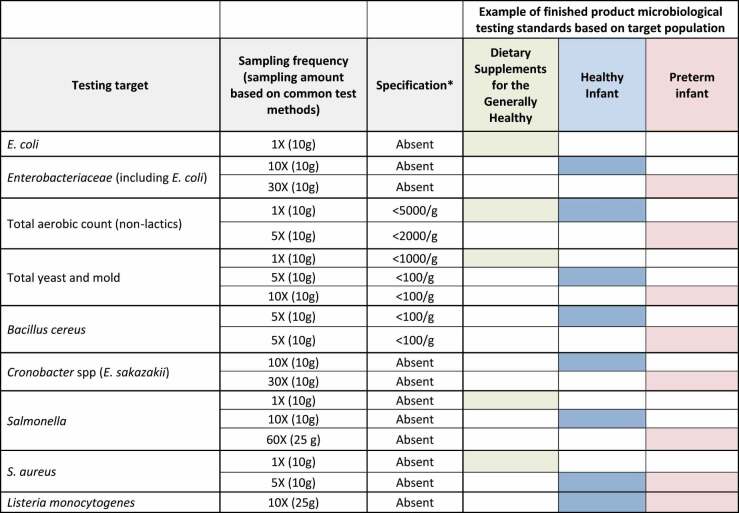

The manner in which the probiotic is given or taken must also be safe. This constitutes safe administration of a properly designed product to the intended host. Safe administration includes an appropriate route of administration to the host and correct manipulation or preparation of the probiotic on site. Products should be delivered at a dose and in a final formulation shown to be safe. Finally, the probiotic must be safe for the host, considering co-morbidities and underlying health concerns. These concerns extend beyond the proper formulation and production. For example, one such administration concern is the potential for cross-contamination of the hospital environment and of vulnerable patients once the probiotic is opened and used on site.^18^ Mixing a dried probiotic in hospital rooms has led to infection of intravenous catheters.^19^ The PROPATRIA study raised the concern that naso-jejunal administration may be contraindicated for some formulations administered to critically ill patients.^20^

Surveillance systems that facilitate both the reporting of AEs per doses administered and the removal from hospital formularies of probiotic product formulations that fail to fully identify the probiotic microorganism (genus, species, and strain) and potency through the end of product shelf-life would enhance the safety of probiotic consumption. In addition, education of providers of medical, nutrition, and healthcare information about the potential risks and benefits of the administration of a probiotic product in individual situations could facilitate shared decision-making regarding the use of probiotic products.^17,21,22^

## Acute risks

### Assessment of microbiota composition or function alterations

Microbiome profiling is the process of assessing baseline microbiota composition and community structure, as well as transcriptional and metabolic readouts. Assessing microbiome outcomes before and after probiotic intake may point to a causal role of probiotics in shaping the microbiome.^23, 24^ Such research may potentially inform hypotheses regarding microbial mechanisms and pathways that promote health, raise safety concerns or determine characteristics of responders and non-responders to probiotic therapy.^24, 25^ Such profiling may have a role in explaining or to a certain degree, predicting, the capacity of probiotics to confer beneficial effects on specific hosts.^25^

These microbiome profiling studies are only occasionally performed in probiotic research. For example, several profiling studies have revealed that intrinsic microbiome features determine probiotic engraftment in microbial communities of consumers.^26^ This suggests that microbiome assessment may provide a tool to tailor probiotic therapy and optimize clinical outcomes. This was observed in a study of *Lacticaseibacillus rhamnosus* GG supplementation of children with cystic fibrosis, where bifidobacteria-dominated fecal microbiota were associated with better clinical outcomes.^27^ In another study, probiotics promoted microbiota maturation, which was associated with reduced sepsis and inflammation in preterm infants.^28, 29^

An association between microbiome features and AEs has not been hitherto described, mostly owing to the paucity of research or inadequate harms reporting.^30^ We therefore recommend that microbiome profiling not be a required component of safety assessment for probiotics. However, such analysis is encouraged, as it may be useful for illuminating determinants of inter-individual differences in response to probiotics and for testing mechanisms and hypotheses.

### Horizontal transfer of antibiotic resistance genes

Since the term probiotic entered the regulatory lexicon via the FAO/WHO expert consultation^31^ and guidelines,^32^ special attention has been paid by regulators, industry, and researchers to the risk of horizontal transfer of AR genes from probiotics to potential pathogens in the gut. *In vivo* transfer via conjugation of AR genes from viable probiotic bacteria acting as donors was documented in 1998^33^ and several papers since have confirmed the transferability of AR genes within the gut for a recent review see.^34^ In general, attention is focused on critically important antibiotics as indicated by the World Health Organization.^35, 36^

Assessing the risk of existing AR genes within probiotic genomes requires both genotypic and phenotypic approaches. The phenotypic approach requires assessment of AR genes expressed by a strain as determined by minimum inhibitory concentration techniques.^37–39^ In some cases, the expression of an AR gene is intrinsic to the species and not due to the expression of genes that can be acquired.^40, 41^ The vancomycin “insensitivity” present in heterofermentative lactobacilli is an example of such intrinsic resistance.^42^ Such resistance is not a safety concern. However, the basis of any phenotypic resistance that is outside the norm for the species must be investigated further. Normal AR ranges for species of common probiotics have been established.^37, 39, 43, 44^ However, when developing strains of species for which such information is not known (next-generation strains), further study is required.

The genotypic approach requires complete genome sequencing (including plasmids) and identification of any known AR genes from the sequence. In general, if those genes are flanked by mobile elements or are plasmid-encoded, the strain should not be commercialized,^12^ but this prohibition may also depend on the antimicrobial in question, its current clinical utility and a risk/benefit analysis for the strain. When AR genes are located in the chromosome and do not have any genetic features that would suggest they are mobilizable, safety must still be carefully considered. If the resistance is for an antibiotic that is not deemed clinically relevant,^35^ it poses low risk. However, if the gene encodes non-intrinsic resistance to an antibiotic that is of clinical importance,^35, 36^ such as vancomycin for example, then the prudent approach would be to not commercialize that strain, unless further consideration determined that it was safe.

The regulatory approach to the tetracycline resistance gene, *tet*(W), in *Bifidobacterium animalis* subsp. *lactis* BB-12 is an example of such a case. The U.S. Food and Drug Administration (FDA), when informed of the presence of this gene, did not question the conclusion that its presence was neither clinically nor environmentally relevant and determined that it did not impact the strain’s status of generally recognized as safe for use in infant formula.^45^ This was based on the absence of a likely mechanism for transfer, the pervasiveness of tetracycline resistance among microbes in general, and the lack of clinical use of tetracycline in infants in the United States. Furthermore, in 2011 the European Food Safety Authority commented about the presence of the *tet*(W) gene in *B. animalis* subsp. *lactis* and concluded that there was no new information that would require a modification of the subspecies’ qualified presumption of safety status.^11^

Even in the absence of risk of conjugal transfer of non-intrinsic AR genes, there is also a possibility of transfer via transformation of naked DNA^46, 47^ or transduction via phage.^48^ Transformation is possible from DNA from non-viable cells, such as cells that have died during production or storage. This concern for dead microbes being a source of AR genes is reflected in the regulatory approach to safety for a postbiotic, which comprises non-viable but beneficial microbes.^49^ The European Food Safety Authority^50^ adopted a precautionary approach in the evaluation of a pasteurized strain of *Akkermansia muciniphila* as a novel food.^51^ The evaluation panel stated: “According to the applicant, the ﬁndings suggest that the strain does not harbor any AMR [antimicrobial resistance] genes of concern.” This was based on an *in-silico* interrogation of AR genes in the genome of the novel strain for AR genes included in the Comprehensive Antibiotic Resistance Database^52^ and National Database of Antibiotic Resistant Organisms.^53^

When considering the safety of AR genes in the genome of probiotics, several issues should be considered: (i) are the genes associated with genes that provide a likely mechanism for horizontal transfer; (ii) are the genes capable of functional expression if transferred to a naïve host; (iii) is the resistance phenotype for the antibiotic typical for the species (intrinsic resistance); (iv) is the resistance phenotype so widespread that the probiotic would not substantively contribute to its presence among environmental microbes; and (v) is the resistance to a clinically relevant antibiotic. These issues must be considered as part of a rigorous analysis that balances benefit with patient or consumer safety and public health concerns. The analysis must consider if the probiotic is being developed for broad distribution to generally healthy consumers (foods or supplements, for which a reasonable certainty of no harm is the general standard) or restricted distribution for patient populations (drugs). Since probiotics have the potential to exacerbate^54^ or mitigate^55^ the reservoir of AR genes harbored in humans, a case-by-case approach to safety is likely needed. Previous authors have suggested systematic approaches to considering this issue.^12,, 14^

Risk assessments for AR genotypes or phenotypes in probiotics are evolving, accommodating rapidly progressing science. The judgments on how to consider phenotype vs genotype or gene presence vs gene transferability can be expected to change based on research findings. Further, genetic modification methods may be useful to modify or eliminate the resistance elements of concern. Depending on the technology employed, this option may be suitable for probiotics for food applications (e.g., plasmid curing) or for next-generation probiotics that may be commercialized via the drug pathway.

We therefore are aligned with current recommendations that all probiotic strains should be screened for AR phenotypes and genotypes. The basis of any phenotypic resistance that is outside the norm for the species must be investigated further. If genes are harbored for antibiotics that are declared critically important by the World Health Organization,^35, 36^ the probiotic should be subjected to a rigorous risk/benefit analysis that balances patient and consumer safety as well as public health concerns. The potential of genetic transfer via transformation poses a low risk, which nevertheless warrants further study. Manufacturers of historical strains that may not have undergone this approach to AR risk assessment should reevaluate strains to assure compliance.

In the context of understanding the risk that probiotic-borne AR genes pose, it should be noted that this caution is not extended to most wild strains of food fermentation microbes, which are consumed live or dead in great numbers in the diets of many populations.^56^ Equally, fecal microbial transplants are not subjected to any AR gene-related restrictions despite the near certainty that many mobilizable AR genes are present in every fecal preparation. Lactic acid bacteria, which are used in the manufacture of fermented foods, have been shown to harbor AR genes.^43^ Horizontal transfer of AR genes from environmental microbes is also a possibility.^57^ The presence of AR genes in these microbes is largely unknown, yet there is no regulatory or scientific call to restrict the consumption of fermented foods on this basis.

### Invasive infection

On rare occasions, probiotics may translocate from the gastrointestinal tract, resulting in invasive infection. A systematic review was conducted of sepsis, bacteremia, and fungemia associated with probiotic administration in children between 1995 and 2021. Of the 49 invasive infections reported, sepsis was most common. The majority of the children meeting the clinical definition of sepsis were under two years old and had a predisposing condition such as prematurity or an indwelling intravenous catheter, and 94% were treated successfully with antimicrobial therapy.^58^ The actual frequency of probiotic-related invasive infection is difficult to determine for multiple reasons. Most of the published case reports occurred outside the context of clinical trials, thus the number of patients receiving probiotics without adverse effects is unclear. Not all clinical laboratories routinely culture and identify probiotic organisms from blood cultures, and harms reporting in probiotic trials often is incomplete.^30^ Nonetheless, culture-proven invasive infection is studied as a primary or secondary outcome in most trials that administer probiotics to preterm neonates, and network meta-analyses suggest that probiotic administration does not increase rates of sepsis in this vulnerable population.^59^ However, the true frequency of probiotic-associated sepsis remains uncertain. We note that Hempel and colleagues^5^ concluded, “Across studies, there was no indication that critically ill and high-risk participants taking probiotics were more likely to experience AEs than control participants with the same health status.” We therefore conclude that invasive infections and sepsis should be monitored diligently in clinical settings and reported fully in all probiotic trials. Using strain-level molecular techniques, clinical isolates should be compared to the administered probiotic. A molecular match of supplemented probiotic microorganism(s) to invasive clinical isolate(s) supports an association between probiotic and systemic infection. Manufacturers should identify and publicize the antibiogram of each commercialized probiotic strain, providing an empirical course of treatment if needed. When assessing the safety of any next-generation probiotic, the potential for translocation should be determined and risks weighed against benefit.

### Probiotic impact on drug function

The gut microbiota can have both direct and indirect effects on the metabolism of drugs, with consequences for both efficacy and toxicity.^60, 61^ For example, it has been known since the early seventies that the urinary excretion^62^ of total sulfanilamide in rats receiving the azo drugs Prontosil or Neoprontosil orally is reduced through the action of microbial azoreductase when the rats are treated with antibiotics,^63^ illustrating that the drug activation can be mediated by the microbiota. Other important microbiota-driven drug^64–66^ metabolisms have been described,^65^ such as decarboxylation (L-dopa^67^), sulfation (acetaminophen^65^) dehydroxylation (caffeic acid and L-dopa^65^), demethylation (methamphetamine^65^), dehalogenation,^68^ and acetylation/diacylation (salicylic acid to form aspirin^65^). Drug-related toxicity can also be reduced by the microbiota. A well-known example is glucuronidation,^65^ a conjugate hydrolysis reaction that links glucuronic acid to a substrate by an UDP-glucuronosyltransferase into hydrophilic and negatively charged glucuronides.^69^ Many anaerobic bacteria can induce β-glucuronidases, an enzyme able to deconjugate xenobiotics and endogenous compounds detoxified earlier via the glucuronidation pathway. This deconjugation can enhance enterohepatic recirculation of toxins, hormones, and various drugs as well as the formation of local carcinogens. Excess amounts of the β-glucuronidases may therefore increase the risk of colon cancer development. However, a certain amount of β-glucuronidase activity is important to guarantee enterohepatic recirculation of essential compounds such as vitamin D, thyroid hormone, or estrogen.

The ability of probiotics to impact drug function may have safety consequences. A relatively new discipline, toximicrobiomics or pharmacomicrobiomics, studies the interactions between the microbiota and xenobiotic compounds.^70,, 71^ Examples such as described by Dikeocha et al.^71^ indicate that it might be important to understand the interplay of microbiota diversity, diet and drug disposition and response and how this may impact future personalized medicine.^72–74^

Research is needed to identify drug-modifying enzymes and to confirm the relevance of these enzymes *in vivo*. The presence of such enzymes in a probiotic and evidence of *in vitro* functionality does not prove these activities would occur in a host. Further, evidence that such enzymes act to an extent that would hamper drug efficacy prior to the drug being absorbed is needed. The nascent nature of this research suggests that it is too early to make specific recommendations. Research focused on developing screens for the presence of enzymes that might metabolically affect specific drugs and databases indicating the related genomic sequences would move this field forward. The end goal would be to identify probiotics that encode enzymes of concern to be able to advise about probiotic – drug incompatibility.

## Long-term risks

### Long-term colonization

The efficacy of a probiotic microbe is not dependent on an ability to colonize the host long-term. Long-term colonization is normally taken to mean that an acutely administered microbe is still detectable from the host weeks or months after dosing has ceased. For an orally administered probiotic, this would mean that the microbe must be actively replicating and that it has established an ongoing presence within the host. Evidence accumulated to-date on recovery of probiotics from feces indicates that most current probiotics do not colonize.^75,, 76^ This almost certainly results from an inability to compete with the resident microbiota. In 1934, the famous Dutch botanist and microbiologist Baas Becking stated, “Everything is everywhere, but the environment selects.” cited in.^77^ In accordance with this concept, in most individuals simply encountering a microbe does not result in colonization, but if an ‘open niche’ is available, an externally applied microbe may be able to fill that niche and colonize.

Research interest has grown to understand what benefits could be provided by strains that are “native” or adapted to the host (as opposed to adapted to growth in large-scale production fermenters, food fermentations, or surviving while sitting on the shelves of traditional retail outlets). Indeed, efforts are underway to achieve better persistence of probiotic strains in human and other hosts using several methods. These efforts include manipulating host diet,^78^ adding prebiotics to support the strain’s growth,^79,, 80^ more precise targeting for adapted strains using computational techniques,^81^ and dose optimization.^82^ Such approaches have led to a new wave of strains that are showing longer term persistence in the host compared to previously published strains. While the array of benefits that are provided by these strains is outside of the scope of this review, this type of capability raises questions for scientists, consumers, and regulators on the long-term safety of these strains. There are now several examples, discussed below, of data in humans demonstrating longer-term engraftment in some study participants than previously shown possible with traditional strains, and this persistence was largely accomplished using autochthonous strains with provenance and physiological adaptations suggesting they are part of the normal human microbiota.

An early example of this phenomenon was the discovery that *Bifidobacterium longum* subp. *longum* AH1206 persisted for 6 months in some adults administered the strain.^83^ Likewise, *Bifidobacterium adolescentis* IVS-1 colonized for 4 weeks post-administration in some adults.^80^ A mixture of *A.muciniphila, Clostridium beijerinckii, Clostridium butyricum, Bifidobacterium longum* subsp. *infantis* and *Anaerobutyricum hallii* given to adults showed all strains persisted in some subjects to varying degrees for 4 weeks post administration.^84^ Evidence suggests *B. longum* subp. *infantis* EVC001 persisted in infants that were fed the strain for approximately one year after administration of the probiotic was discontinued.^85, 86^ *Lactiplantibacillus plantarum* ATCC 202,195 administered to infants appeared to persist for nearly 6 months based on analysis of plated isolates.^87^ Four out of a mixture of five probiotic strains in a commercial product were able to persist for several months post-supplementation when administered to premature infants.^29^

Persistent probiotics are also not limited solely to gastrointestinal niches and digestive health applications. Several vaginal strains including *Lactobacillus crispatus* CTV-05,^88^ *L. rhamnosus* GR-1, and *Limosilactobacillus reuteri* RC-14,^89^ all persist in the vaginal environment on the order of weeks to months. Administration of *B. animalis* subsp. *lactis* HN019 for 30 days resulted in its recovery for at least 60 days from the gingiva.^90^ Several commercial efforts are underway to identify and test colonization of the skin microbiota with probiotic strains adapted for that environment.^91^ These examples demonstrate the direction new probiotic strain discovery is taking. Strategies specifically aimed at developing long-term colonizing probiotics should take a risk-benefit approach.

What are the safety concerns? In most instances colonization with a probiotic derived from the common commensal microbiota at low levels should not be problematic to host health. Even high levels of a microbe with no obvious virulence potential should not negatively impact host health. However, it is conceivable that there could be risks associated with the increased exposure inherent to long-term colonizing probiotics. Potential risks include: (i) The probiotic could displace a microbe performing an important function; (ii) the probiotic could negatively impact the structure and function of the surrounding microbiota; and (iii) if the normal gut barrier is breached, a probiotic could access the systemic circulation, resulting in invasive infection. This last example was observed in a small but significant increase in *Lactobacillus* bacteremia in intensive care unit patients receiving probiotics in a Boston hospital, albeit this was not linked directly to colonization of the probiotic.^92^ Lack of probiotic colonization allows the prescribing physician or consumer to retain control of the dosing regimen, rather than depend on a self-replicating probiotic. Knowing the antibiotic sensitivity profile of a particular probiotic provides a strategy for eradication, if needed, and thus reduces the risk of long-term colonization in the unlikely case such risks should become apparent.

What are the potential benefits? One could make an argument that the long-term presence of the probiotic, which by its definition imparts a health benefit, could be an efficient and effective way of delivering long-term health benefits. Indeed, a microbe that can permanently occupy a vacant niche and provide a missing metabolic function – such as the ability to metabolize human milk oligosaccharides in an infant – that contributes to host health could represent an excellent probiotic candidate. Another consideration is that if the niche is destined to be occupied, would a long-term colonizing probiotic be a safer, more desirable occupier than an unknown microbe?

This new wave of strains that appear better adapted for establishing a presence and living in the human body has potential for achieving distinct and superior benefits. For example, imparting enzymatic capacity to compensate for a metabolic disorder such as phenylketonuria could be best achieved by a long-term colonizing probiotic. With our current understanding, we recommend that the development of a long-term colonizing probiotic be done only with a clear objective of achieving benefits not easily, reliably, or economically attainable otherwise and with weighing the risks against those clearly defined benefits. We recommend that careful consideration be dedicated to determining what long-term safety data might be relevant to probiotic strains that persist in the host. Research should be conducted to determine relevant acute exposure tests and biomarkers that are useful for assessing safety of long-term colonizing probiotics.

### Assessment of microbiota composition or function alterations

Microbiome assessments conducted in conjunction with probiotic intervention trials have been undertaken primarily to address microbiota-mediated mechanisms driving efficacy, not as a measure of safety. But recent studies showing that a certain blend of live strains delayed microbiota composition recovery after antibiotic treatment highlights the potential role that microbiota readouts might have in addressing long-term safety. This study showed that humans and mice treated with antibiotics and then a probiotic mixture comprising 11 strains delayed for at least 6 months the recovery of gut mucosal and fecal microbiome composition and function to previous states compared to their counterparts, who did not receive probiotics.^93^ In fact, their gut microbiomes assumed a new steady state during probiotic administration and thereafter, which was significantly divergent from its baseline, although the possible benefit of this divergence was not considered. This study and others also demonstrated a lower number of observed species in the gut microbiota of probiotics-treated hosts after antibiotics.^93–95^ While these findings can be potentially harnessed to remodel the microbiota to a more desirable configuration, they might also raise a safety concern as microbiota alterations and reduced alpha diversity may be associated with infectious, inflammatory, and metabolic sequelae.^96^ Furthermore, a *post-hoc* analysis of the previous study revealed that probiotics administered after antibiotics caused an expansion in antibiotic resistance genes within the mucosal microbiota, in particular to vancomycin; however, this in-silico finding was not confirmed *in-vivo*.^54^ Vancomycin resistance manifested as an expansion of the vanG and vanSD genes (in humans and mice, respectively). The probiotic species were not the source for these genes, but came from the bloom of some members of the microbiota, specifically *Clostridium, Blautia*, and *Romboutsia*, which were carrying those genes. Taken together, there is evidence that the probiotic blend administered in this study given after antibiotic treatments alters the microbiota structure and function. However, there is no clear link between these alterations and clinical AEs nor is there evidence that this observation can be extended to other probiotic preparations. We therefore do not recommend that microbiota community composition and function analyses be routine for probiotic safety assessments, but recognize that profiling of microbiota structure and function may be useful for testing mechanisms and hypotheses. Research is needed to understand the clinical implications of any observed microbiota structure of function alterations.

## What long-term safety studies are indicated?

Long-term safety studies for probiotics are much less well defined than those addressing acute safety issues. For drugs and biologics, subacute and chronic toxicities are important for revealing any detrimental effects associated with repeated dosing and/or associated bioaccumulation that may occur. In our context, bioaccumulation can refer to microbial metabolites but also microbial proliferation and colonization. These types of studies are usually conducted in animal models using validated procedures designed to provide insight into risk for humans. These studies are inherent to the drug development process prior to clinical use.^97^ Using the drug development pathway for FDA as a guide, this issue of long-term safety generally falls into a post-market monitoring system upon agreement with the manufacturer for submitting safety updates along with voluntary consumer and physician reporting of AEs to MedWatch.^98^ Long-term (estimated at >6 months) safety trials in humans may not be required for drug approval, although this would be assessed on a case-by-case basis. For example, most approved biologics for ulcerative colitis have been approved in recent years with little long-term data and some conflicting safety data. A literature review in this area revealed such studies have follow-ups that range from 16 to (at most) approximately 24 months stratified by collecting reports of AEs and serious AEs while monitoring and connecting to various other outcomes.^99^ Probiotics, which are often sold as supplements and not as drugs (live biotherapeutic products), lack the rigorous reporting requirements for post-market safety, although a formal process for reporting problems with dietary supplements exists. In the United States, MedWatch provides health professionals, patients, and consumers an avenue to report safety concerns for FDA-regulated product.^98^ Few clinical trials on probiotics follow study cohorts long-term, leaving a gap in long-term data.

Based on the traditional toxicological designs used to assess the safety of small molecules, long-term chronic studies in animal models are defined typically as 6 months to 1 year in duration with *a priori* experiential knowledge or predisposition toward affected organs, reversibility of toxicity, (no) observed effect levels, and quantifying clinical risk at expected dose for long-term treatment.^100, 101^ Translating this preclinical framework to next-generation live biotherapeutics, which lack history of safe use, would suggest that 12-month studies in humans would be in line with expectations for new molecular agents/targets when little-to-no post-market experience is available and should potentially be conducted concurrently with Phase III clinical trials. However, most of the established toxicological frameworks have been developed over the years from pharmacokinetic/toxicokinetics and a variety of preclinical testing modalities. However, their relevance to probiotics that do not generally enter the bloodstream or exhibit traditional small-molecule decay rates is not clearly apparent.

Given this backdrop, the safety challenge is defining relevant outcomes and pathways that may span the cadre of intended organisms and their metabolic potentials within a microbial community setting of the gut.^102^ This becomes a confounding proposition given our current limited understanding of the microbiota, colonization, functional parameters, and their contribution to health and well-being. Predictive biomarkers of health and disease based on microbiota community structure and function are needed.^102^ Some relevant features might include thresholds affecting diversity indices and/or microbial community alterations that indicate concern for reflexivity to normalcy or community structures indicative of, for example, pro-inflammatory, pro-obesity, disrupted immune homeostasis, metabolic syndrome/diabetes dispositions or other additional conditions that may require basic research to resolve validated metagenomic markers.^102,103^ Published time-course studies and follow-up in study subjects would provide some basis for establishing the validity and usefulness of such parameters and whether they are generalizable across populations, thereby contributing to the development and continuing innovation of live biotherapeutic products. However, regulatory requirement prior to approval process in this regard may overreach without rational basis or may simply be unachievable with currently available tools.

It is clear that the state of science and understanding of key metrics for safety in specialized population or disease states (and associated model systems) have not yet achieved a literature base or consensus for establishing long-term safety recommendations for probiotics. Adding to this uncertainly is the potential for rationally designed microbial consortia with functional properties that are synergistic. As we learn more about the ways in which the microbiota impacts human physiology in the long-term, specific approaches to long-term follow-up may become warranted.

Based on current FDA requirements for biologic drugs, including fecal microbial transplants, we do not recommend specific tests or length of follow-up to address long-term issues. Research in humans focused on determining if a probiotic changes the long-term microbiota composition or function should include collecting data on AEs, similar to acute studies. We advise research into animal models, especially as applied to next-generation strains, to further our mechanistic understanding of how to measure potential long-term effects. In accordance with regulatory requirements for foods, supplements or drugs, companies must track and report adverse events.

## Vulnerable target populations

Long-term studies designed to demonstrate probiotic safety in populations at risk (such as individuals with weakened/impaired immune function, aged people, newborns, particularly preterm infants) are scarce. Although beneficial effects of probiotics in such groups are reported, immunocompromised hosts might be at higher risk for AEs due to their reduced ability to defend against a microbial challenge.^104^ Tracking the long-term impact for weeks or months of probiotics administered through infant formula from birth is warranted. The homogeneous diet and developing gut microbiota may enable such exposure to probiotics to permanently impact the development of their microbial ecosystem.

Evidence from short-term observations suggest that certain probiotic strains might behave as opportunistic pathogens in populations who are immunocompromised, stressed, aged, or newly born.^105^ AEs include life-threatening pneumonia,^106^ endocarditis,^107–111^ and sepsis.^112–115^ In general, it has been suggested that in vulnerable populations, the presence of a single major risk factor, such as immunocompromised state, or more than one minor risk factor, merits caution in using probiotics.^116^ However, to the extent compelling evidence exists that probiotics can benefit some vulnerable populations, their use should be considered. Based on the available data, extra monitoring is warranted when probiotics are administered to vulnerable target populations.

Risks of probiotics to pregnant and lactating women have been reviewed.^117–119^ Of 100 eligible studies of probiotic administration during pregnancy, only 28 reported AEs. Of these, only 11 reported AEs that potentially could have a causal relationship with the treatment, including gastrointestinal problems, nausea, and headache; but no serious health concerns were reported for mother or infant.^117^ The remaining 72 studies did not report AEs. One study reported an increased risk of vaginal discharge and changes in stool consistency when administering *L. rhamnosus* GR-1 and *L. reuteri* RC-14,^118–120^ but a recent study did not confirm this observation.^121^ The reviewed publications suggest that probiotics do not appear to pose a safety concern for this population. However, the findings of a Cochrane review^122^ showed an increased risk of pre-eclampsia when data from four trials in obese women – who are at an increased risk for pre-eclampsia – were pooled (31 cases of pre-eclampsia in 472 women who took probiotics versus 17 in 483 women in the placebo groups). Although the data are not robust, we still recommend that probiotics for mild to morbidly obese pregnant women be administered only with concomitant monitoring for potential risk of pre-eclampsia.

Some concerns have been expressed regarding metabolic activity of some probiotics used in certain populations. D-lactic acidosis can occur in people with surgically altered gut anatomy, such as short bowel or bariatric surgery, resulting from activity of resident microbes.^123^ There are few published accounts linking this to probiotic supplementation.^124^ Lack of data in preterm infants led to a caution about using D-lactate-producing probiotics in this population,^125^ although a controlled trial in healthy full-term infants showed that D-lactate-producing *L. reuteri* DSM-17938 did not result in acidosis^126^ and probiotic-containing infant formulas were not associated with acidosis.^127^

Preterm infants warrant special consideration given the unique window of opportunity for modulation of microbiota structure and function amidst numerous competing prenatal, perinatal, and postnatal factors.^128^ Neonatal microbiota-targeting therapies have the potential to influence host biology throughout the lifespan, either by introducing allochthonous microbial strains when conditions may be most permissive to colonization, or by influencing early developmental trajectories of vital organs including the brain.^129^ To date, there is little evidence to suggest that early-life probiotic supplementation adversely influences neurodevelopmental outcomes. In one follow-up study of 1,099 very preterm infants, there was no difference in major neurodevelopment outcomes at 3–5 years of age in surviving infants who had received compared to those who had not received probiotics. Intriguingly, deafness was less prevalent in the probiotic-treated children, and this could not be attributed to differences in the numbers of courses of antibiotics or to the total days of vancomycin or gentamicin received.^130^

Other studies have identified a link between early-life microbiota alterations and obesity. A large cohort study of 333,353 children in the United States reported that prescriptions of antibiotics and acid-suppressive medications in the first two years of life are associated with obesity later in childhood; these associations were strengthened with each additional class of these microbiota-altering drugs and with each additional 30-day prescription.^131^ Similarly, exposure to household disinfectants early in life is associated with higher body mass index at three years of age in a cohort of 757 Canadian infants.^132^ Importantly, studies evaluating rates of childhood obesity following probiotic therapy in the perinatal and infant period have not reported any AEs with respect to body mass index.^133, ,134^ Thus, the limited evidence available does not suggest that early-life probiotic use increases the risk of adverse outcomes in childhood. There are not enough data to determine whether potential associations may exist between perinatal probiotic use and AEs in adulthood. **Therefore, we encourage a minimum of 2-year (when most outcomes are no longer corrected for prematurity) follow up from studies of premature infants who received probiotics or not in the perinatal period to compare metabolic, allergic, immune, and other health outcomes.**

## Probiotic quality considerations

In general, high quality, safe probiotic products are produced under dietary supplement and food regulations, although there have been incidences of noncompliance documented for supplements^15^ and calls for improved product quality.^135^ Dietary supplements are a category of products developed to supplement the diet of the generally healthy population, not to treat or prevent disease. This is important because while the safety standard for dietary supplements requires that they will reasonably expected to be safe under the labeled conditions of use, they do not need to be established as safe for more vulnerable patient populations.^9^ The intended use is crucial in establishing essential efficacy and safety requirements. In different jurisdictions, various guidelines and regulations exist for good manufacturing practices. In the United States, the Food and Drug Administration distinguishes, among others, current good manufacturing practices for food and drugs:

- For food and dietary supplements: These describe the “methods, equipment, facilities, and controls for producing processed food”. These are meant to ensure that the food is safe to eat.^136^
- For drugs: These assure “the identity, strength, quality, and purity of drug products by requiring that manufacturers of medications adequately control manufacturing operations”.^137^

The onus is on manufacturers to establish appropriate product specifications based on intended use and acceptable risk levels. Reputable manufacturers establish rigorous purity, potency, and identity quality standards consistent with the intended population and sufficient for that use. These standards require quality control and quality assurance protocols.

Quality specifications sufficient for vulnerable populations can be developed.^138^ Testing requirements more stringent than those sufficient for healthy populations can be requested from product manufacturers (Table 1). Adherence to these standards can be verified independently by a third party with this expertise.^135^ Even if adherence to these higher standards is not referenced on product labels, certificates of analysis should specify testing results. Hospital pharmacists who stock formularies and scientists who aim to investigate probiotics in these vulnerable (patient) populations should work with the supplier to provide extra product testing and visibility of those results. Pharmacies can negotiate quality agreements with vendors that would delineate their expectations for the strains present, their potency, the limits for contaminants (microbiological and others), and other criteria. This agreement could also mandate that any product change – as defined in the agreement – would require the vendor to notify the pharmacist or researcher. Such an agreement would increase the burden on a hospital pharmacist or an academic investigator but would establish the testing standards a pharmacy would expect of the products it stocks. A sophisticated dietary supplement provider should be able to assure the hospital formulary and investigator of their product quality and the process they have followed to assure and control this.

We recommend that probiotic dietary supplements targeted for vulnerable populations undergo third-party verification of product quality (purity, potency, and identity), which are accurately communicated on product labels. Further, such products should undergo testing to meet quality standards appropriate for that population. Direct agreements with probiotic manufacturers may be useful for developing products to meet stricter quality control standards. We also recommend that approaches to product labeling be reviewed to enable stricter standards to be communicated on product labels.

## Toward progress in reporting adverse events

Reporting AEs in randomized controlled trials is critically important to understanding the trade-offs between the benefits and harms of interventions. Missing or insufficient reporting of harm-related data is not specific to the probiotic field. Similar issues have been reported regarding other interventions.^139–142^ Still, clinical researchers should follow long-standing recommendations for reporting harms as delineated in the Consolidated Standards of Reporting Trials Group published guidelines for complete and detailed reporting of harms, known as the CONSORT Extension for Harms.^143^ Authors should provide a balanced discussion of the benefits and harms. We recommend that in all clinical trials on probiotics, data on adverse events should be collected. Events should be listed and defined, with reference to standardized criteria where appropriate. For each study arm, the absolute risk of each adverse event, using appropriate metrics for recurrent events, and the number of participants withdrawn due to harms should be presented. We also recommend that the authors should provide a balanced discussion of the benefits and harms.

## Conclusions

We met to identify emerging acute and long-term risks associated with probiotics and to update recommendations pertaining to probiotic safety. Probiotic safety encompasses properties inherent to the probiotic, to the consumer/patient, and to the manufacturing process (contaminated probiotics products represent a safety concern). Table 2 summarizes our recommendations. Some should be implemented promptly but others warrant further study before actionable recommendations can be developed.

**Table 2.** Summary of key recommendations regarding acute and long-term safety concerns for probiotics. AR, antibiotic resistance.

Safety risks	Recommendation
**ACUTE SAFETY**	
Microbiome assessments	Microbiome profiling is not a required component of safety assessment for probiotics. Microbiome profiling is encouraged, as it may be useful for illuminating determinants of inter-individual differences in response to probiotics and for testing mechanisms and hypotheses.
Antibiotic resistance	All probiotic strains should be screened for AR phenotypes and genotypes. Any phenotypic resistance that is outside the norm for the species must be investigated further. If genes for antibiotics that are declared critically important by the World Health Organization (see text) are harbored, the probiotic should be subjected to a rigorous risk/benefit analysis that balances patient and consumer safety as well as public health concerns. The potential of genetic transfer via transformation poses a low risk, which nevertheless warrants further study. Manufacturers of historical strains that may not have undergone this approach to AR risk assessment should re-evaluate strains to assure compliance.
Invasive infection	Invasive infections and sepsis should be monitored diligently in clinical settings and reported fully in all probiotic trials. Using strain-level molecular techniques, clinical isolates should be compared to the administered probiotic. A molecular match of supplemented probiotic microorganism(s) to invasive clinical isolate(s) supports an association between probiotic and systemic infection. Manufacturers should identify and publicize the antibiogram of each commercialized probiotic strain, providing an empirical course of treatment if needed. When assessing the safety of any next-generation probiotic, the potential for translocation should be determined and risks weighed against benefit.
Probiotic:drug compatibility	Research has shown that probiotics encode enzymes that can interact with some drugs. Additional research is needed before recommendations can be made.
Vulnerable populations	Probiotics for mild to morbidly obese pregnant women be administered with awareness of an increased risk of pre-eclampsia. Extra monitoring is warranted when probiotics are administered to vulnerable target populations.
**LONG TERM SAFETY**	
Long-term studies	Specific tests or length of follow-up to address long-term safety considerations are not required, as is consistent with current FDA requirements for biologic drugs, including fecal microbial transplants. Research in humans focused on determining if a probiotic changes the long-term microbiota composition or function should include collecting data on AEs, similar to acute studies. Research into animal models, especially as applied to next-generation strains, is advised to further our mechanistic understanding of how to measure potential long-term effects. In accordance with regulatory requirements for foods, supplements or drugs, companies must track and report adverse events.
Long-term colonization	Development of a long-term colonizing probiotic be done only with a clear objective of achieving benefits not easily, reliably, or economically attainable otherwise and with weighing of the risks in light of benefits. Careful consideration should be dedicated to determining what long-term safety data might be relevant to probiotic strains that persist in the host. Research should be conducted to determine relevant acute exposure tests and biomarkers that are useful for assessing safety of long-term colonizing probiotics.
Microbiome assessments	Microbiota community composition and function analyses should not be required for probiotic safety assessments. Such profiling may be useful for testing mechanisms and hypotheses. Research is needed to understand the clinical implications of any observed microbiota structure of function alterations.
Vulnerable populations	A follow-up is encouraged for a minimum of 2-year (when most outcomes are no longer corrected for prematurity) from studies of premature infants who received probiotics or not in the perinatal period to compare metabolic, allergic, immune, and other health outcomes.
**GENERAL RECOMMENDATIONS**	
Probiotic quality	Probiotic dietary supplements targeted for vulnerable populations should undergo third-party verification of product quality (purity, potency, and identity), which are accurately communicated on product labels. Such products should undergo testing to meet quality standards appropriate for that population. Direct agreements with probiotic manufacturers may be useful for developing products to meet stricter quality control standards. Product labels would ideally communicate stricter standards.
Adverse event reporting	All clinical trials should rigorously collect and report data on adverse events. Events should be listed and defined, with reference to standardized criteria where appropriate. For each study arm, the absolute risk of each adverse event, using appropriate metrics for recurrent events, and the number of participants withdrawn due to harms should be presented. A balanced discussion of the benefits and harms should be presented.
Probiotic product labeling	Probiotic product formulations should remove probiotic products that fail to fully identify the probiotic microorganism (genus, species, and strain) and potency through the end of product shelf life.

Potential long-term concerns are difficult to address as data are limited. But for probiotics, an approach should align with regulatory approaches used for other biologics. Ongoing research will surely bring to light new long-term safety implications that will need to be considered in safety assessments. But applying biomarkers and other outcomes that are often used in short-term assessments may not be appropriate to address long-term safety implications. For example, if a strain colonizes the host, enhanced long-term evaluations are recommended. However, assessment of microbiome alterations is likely insufficient for this task, since the clinical implications of these alterations are unclear. Research is needed to clarify which types of high-risk groups require closer long-term follow-up. For example, mild to morbidly obese women may need closer monitoring during pregnancy and additional long-term follow-up studies are warranted for premature infants.

No assurance of absence of harm can be guaranteed with any food, supplement, or medical intervention. This paper aims to engender those tasked with assessing safety of probiotics to consider the emerging issues described herein. In some cases, regulatory frameworks notwithstanding, the risk-to-benefit ratio must be considered. We hope this paper helps guide the scientific, regulatory, and medical communities about considerations for this judgment.

## Abbreviations

AE: adverse event
AR: antibiotic resistance
GMP: good manufacturing requirements
MIC: minimum inhibitory concentration
FDA: U.S. Food and Drug Administration
